# AI-driven data-efficient estimation of partition functions in disordered materials

**DOI:** 10.1038/s41598-026-37953-6

**Published:** 2026-03-23

**Authors:** Maciej J. Karcz, Luca Messina, Eiji Kawasaki, Emeric Bourasseau

**Affiliations:** 1 CEA, DES, IRESNE, DEC, Cadarache, F-13108 Saint-Paul-Lez-Durance, France; 2 Université Paris-Saclay, CEA, LIST, F-91120 Palaiseau, France

**Keywords:** Materials science, Condensed-matter physics, Theory and computation, Computational science, Software, Applied physics, Condensed-matter physics

## Abstract

This work presents PULSE (Partition function Unsupervised Learning Sampling and Evaluation), a novel generative approach to efficiently estimate the partition function of disordered compounds, without requiring pre-existing training datasets and at a fraction of the computational cost compared to traditional methods. By enabling targeted calculations of atomic-scale properties, PULSE overcomes key limitations of existing sampling techniques, which either require prohibitive computational effort (Monte Carlo) or result in a partial exploration of the configuration space (Special Quasirandom Structures). The approach, based on an inverse variational autoencoder architecture, can also generate representative configuration sets tailored to specific properties, offering a powerful tool for constructing optimized datasets for training interatomic potentials. We demonstrate the capabilities of our method by computing point-defect formation energies and concentrations in uranium-plutonium mixed oxides. Furthermore, we show that the generated configurations provide physical insight into the role of local environments on defect behavior. Beyond this specific application, PULSE is broadly applicable to studying disorder-driven properties in complex materials, including mixed oxides and high-entropy alloys.

## Introduction

Multi-component compounds play a crucial role across various disciplines, from structural alloys^1^ and semiconductors^2,3^ to energy storage systems^4^ and catalysts^5^. These materials are characterized by complex atomic interactions that often give rise to unique properties, such as enhanced strength, tunable electronic behavior, or improved catalytic activity. However, these compounds can be characterized by different levels of chemical disorder^6^. Such disorder leads to a vast number of possible atomic configurations, making it computationally expensive and time-consuming to explore the entire configuration space, which is particularly relevant in systems like high-entropy alloys (HEAs)^7–9^ or mixed-actinide oxides (MOX) used as nuclear fuels^10^.

The challenge of chemical disorder makes it difficult to study certain properties, such as those related to crystal point defects, which play a crucial role in material behavior, including phase stability driven by atomic transport^11^, fragility^12^, and thermal stability^13^. A common way to access such properties is through the use of Special Quasirandom Structures (SQS)^14,15^, which assume that the configuration space can be represented with few atomic configurations that approximate a random (disordered) solid solution. However, given that only a very limited number of SQS configurations are considered, they may not suffice to accurately represent the entire configuration space. Markov Chain Monte Carlo techniques provide an alternative by sampling a broader range of configurations^15^. However, they require a very high number of configurations and thus significant computational resources^16^. Machine learning approaches, particularly generative models, offer significant potential for exploring properties that depend on large configuration spaces. However, a key challenge lies in the common prerequisite of initial training datasets with high-quality data to generate meaningful outputs, which may not always be readily available.

In this work, we propose a new method called PULSE (Partition function Unsupervised Learning Sampling and Evaluation), where we apply a machine learning model based on variational autoencoders (VAE)^17,18^, referred to as inverse variational autoencoder (IVAE), for targeting the calculation of partition functions in disordered materials. This framework demonstrates high predictive accuracy in studying crystal defect properties in disordered compounds without requiring any initial databases. By using the IVAE model within this approach, we demonstrate how it can predict defect formation energies, concentrations, and the influence of local atomic environments with significantly reduced computational cost compared to traditional methods. Remarkably, it requires orders of magnitude fewer energy calculations than traditional Monte Carlo methods^15^ to accurately predict high-temperature properties. Its capabilities are further highlighted through an application to actinide mixed oxides, demonstrating its potential for complex disordered materials.

The proposed IVAE model operates within the variational inference framework, reversing the roles of encoder and decoder to map simple input distributions (e.g., Gaussian) to complex ones^19–21^. This approach enables self-training without relying on pre-existing data, offering more advantages in terms of flexibility over other methods, such as normalizing flows^22–25^ and autoregressive models^26^. Notably, IVAE does not require input and output dimensions to match, unlike normalizing flows, and allows for more expressive output probability distributions without the constraints of requiring tractable distributions, as is the case with autoregressive models.

## Results

### Targeting the partition function

The partition function $Z_T$ can be used to define the probability of a given configuration $\boldsymbol{x}$ in the following way:

1 $$\begin{aligned} P_T(\boldsymbol{x}) = \frac{{f_T(\boldsymbol{x})}}{{Z_T}}. \end{aligned}$$

Determining $Z_T$ allows for the definition of $P_T(\boldsymbol{x})$. Conversely, knowing $P_T(\boldsymbol{x})$ enables the computation of $Z_T$. In most practical scenarios, $P_T(\boldsymbol{x})$ is an unknown and potentially complex distribution of the target system, making direct computation challenging.

One approach is to find another distribution $P(\boldsymbol{y})$ and use it to obtain $P_T(\boldsymbol{x})$. The idea is that if $P(\boldsymbol{y})$ is chosen to be simple and easy to sample from, and it is possible to connect it to $P_T(\boldsymbol{x})$, then samples $\boldsymbol{y}$ from $P(\boldsymbol{y})$ can be used to obtain $\boldsymbol{x}$ distributed according to $P_T(\boldsymbol{x})$. To achieve this, an arbitrary probability distribution $R_{\boldsymbol{\phi }}(\boldsymbol{x} | \boldsymbol{y})$ is introduced, which can be used to sample $\boldsymbol{x}$ given $\boldsymbol{y}$. However, $\boldsymbol{x}$ obtained in this manner is not necessarily distributed according to $P_T(\boldsymbol{x})$, and its distribution heavily depends on the form of $R_{\boldsymbol{\phi }}(\boldsymbol{x} | \boldsymbol{y})$. To connect $R_{\boldsymbol{\phi }}(\boldsymbol{x} | \boldsymbol{y})$ and $P_T(\boldsymbol{x})$, another conditional distribution $Q_{\boldsymbol{\theta }}(\boldsymbol{y} | \boldsymbol{x})$ is introduced, allowing for the reverse sampling $\boldsymbol{y}$ given $\boldsymbol{x}$. The distributions $P(\boldsymbol{y})$, $R_{\boldsymbol{\phi }}(\boldsymbol{x} | \boldsymbol{y})$, and $Q_{\boldsymbol{\theta }}(\boldsymbol{y} | \boldsymbol{x})$ are assumed to be normalized, satisfying $\sum _{\boldsymbol{y}}P(\boldsymbol{y}) = 1$, $\sum _{\boldsymbol{y}}Q_{\boldsymbol{\theta }}(\boldsymbol{y}|\boldsymbol{x}) = 1$, and $\sum _{\boldsymbol{x}}R_{\boldsymbol{\phi }}(\boldsymbol{x}|\boldsymbol{y}) = 1$. This allows for the following expression:

2 $$\begin{aligned} P_T(\boldsymbol{x}) Q_{\boldsymbol{\theta }}(\boldsymbol{y} | \boldsymbol{x}) = P(\boldsymbol{y}) R_{\boldsymbol{\phi }}(\boldsymbol{x} | \boldsymbol{y}). \end{aligned}$$

Eq. (2) represents an ideal case scenario where the distributions $R_{\boldsymbol{\phi }}$ and $Q_{\boldsymbol{\theta }}$ enable an exact mapping between $P_T(\boldsymbol{x})$ and $P(\boldsymbol{y})$. Since finding an exact solution is often impractical, the objective is to determine $R_{\boldsymbol{\phi }}$ and $Q_{\boldsymbol{\theta }}$ that approximate $P_T(\boldsymbol{x})$ in Eq. (2). This can be framed as an optimization problem, where the task of approximating $P_T(\boldsymbol{x})$ is transformed into finding the optimal set of parameters $\boldsymbol{\phi }$ and $\boldsymbol{\theta }$. The distribution $R_{\boldsymbol{\phi }}$ can be defined using the product distribution proposed in^18^:

3 $$\begin{aligned} R_{\boldsymbol{\phi }}(\boldsymbol{x}|\boldsymbol{y}) = \prod _i \frac{e^{ { I(x^{(i)}) \phi ^{(i)}(\boldsymbol{y})}}}{2\cosh {(\phi ^{(i)}(\boldsymbol{y}))}}, \end{aligned}$$

where $I(x^{(i)})$ is an indicator function, explained further in Sec. 4. A convenient choice for $P(\boldsymbol{y})$ is to define $\boldsymbol{y}$ as a series of independent coin flips, with each $y^{(i)}$ having a probability of 1/2 of being either 1 or −1. For the $Q_{\boldsymbol{\theta }}$ distribution, the same logic as in Eq. (3) can be applied:

4 $$\begin{aligned} Q_{\boldsymbol{\theta }}(\boldsymbol{y}|\boldsymbol{x}) = \prod _i \frac{e^{{y^{(i)} \theta ^{(i)}(\boldsymbol{x})}}}{2\cosh {(\theta ^{(i)}(\boldsymbol{x}}))}. \end{aligned}$$

Both Eq. (3) and Eq. (4) are Bernoulli distributions with logit parameters.

To assess whether a given set of parameters performs effectively, the difference between both sides of Eq. (2) can be measured using the Kullback-Leibler (KL) divergence^27^. Since the computation relies on variational inference without an initial training database, the reverse KL divergence is applied to Eq. (2), $D_{\mathrm{KL}}\left[ P(\boldsymbol{y}) R_{\boldsymbol{\phi }}(\boldsymbol{x} | \boldsymbol{y}) \; \parallel \; P_T(\boldsymbol{x}) Q_{\boldsymbol{\theta }}(\boldsymbol{y} | \boldsymbol{x}) \right]$, leading to:

5 $$\begin{aligned} \ln {Z_T} \geqslant {\mathbb {E}}_{ \boldsymbol{x} \sim R_{\boldsymbol{\phi }},\,\boldsymbol{y} \sim P}\left[ \ln {\frac{f_T(\boldsymbol{x}) Q_{\boldsymbol{\theta }}(\boldsymbol{y}|\boldsymbol{x})}{ P(\boldsymbol{y}) R_{\boldsymbol{\phi }}(\boldsymbol{x} | \boldsymbol{y}) } }\right] = -{\mathcal {L}}(\boldsymbol{\phi },\boldsymbol{\theta }). \end{aligned}$$

$\boldsymbol{\phi }$ and $\boldsymbol{\theta }$ correspond to the parameters of two neural networks that are tuned during the training process. While $R_{\boldsymbol{\phi }}(\boldsymbol{x} | \boldsymbol{y})$ factorizes over sites, correlations are effectively learned by the neural network conditionally on the latent variable $\boldsymbol{x}$.

The function $-{\mathcal {L}}(\boldsymbol{\phi }, \boldsymbol{\theta })$ serves as the lower bound of $\ln {Z_T}$ and can be interpreted as the loss function of the IVAE. The objective is to maximize this function with respect to $\boldsymbol{\phi }$ and $\boldsymbol{\theta }$ to estimate $\ln {Z_T}$. As explained in Sec. 4, computing the loss function allows direct computation of the defect concentration. This approach is similar to established concepts in the literature, such as the mean-field approximation^28^ and the Bogoliubov inequality^29^, where variational methods are used to derive bounds on the partition function and the free energy. The key difference in our case is that the distributions are parameterized using neural networks.

### Unsupervised training loop and functionality of the model

**Fig. 1:**
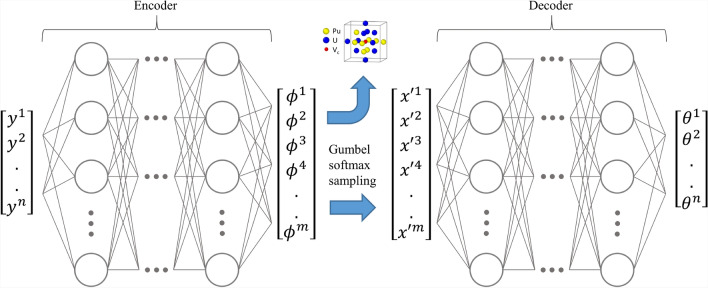
General architecture of the IVAE model presented in this work. Samples from the input $P(\boldsymbol{y})$ distribution are denoted as *y*. $\phi$ corresponds to the predicted parameters of $R_{\boldsymbol{\phi }}$ distribution from Eq. (3). $x'$ are the samples from the Gumbel softmax distribution from the original $R_{\boldsymbol{\phi }}$ and $\theta$ are the predicted parameters of the $Q_{\boldsymbol{\theta }}$ distribution from Eq. (4).

**Fig. 2:**
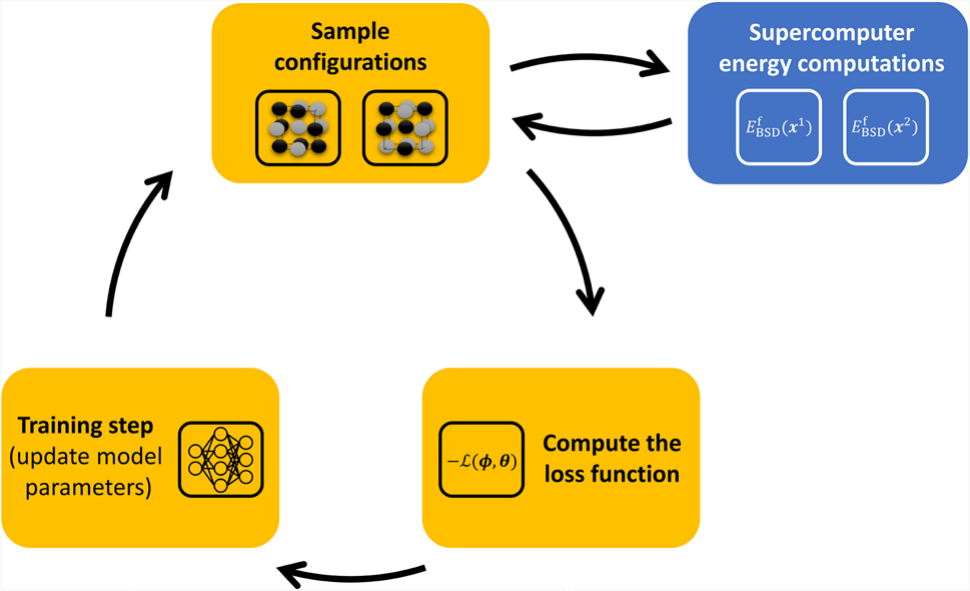
Schematic visualization of the PULSE unsupervised training loop. Initially, the model parameters are randomly initialized. At each iteration, $\boldsymbol{y}$ is sampled from $P(\boldsymbol{y})$, then atomic configurations $\boldsymbol{x}$ are sampled from $R_{\boldsymbol{\phi }}(\boldsymbol{x}|\boldsymbol{y})$, the loss function is computed, and the model weights are updated. This process is repeated until convergence.

The general architecture of the model within the PULSE framework is shown in Fig. 1. Maximizing the right-hand side of Eq. (5) ensures that the predictions of the model are always less than or equal to the true logarithm of the partition function, thereby transforming the estimation into an optimization task that facilitates the training process. The model is trained using an unsupervised loop, as schematically represented in Fig. 2. In each iteration, it samples $\boldsymbol{y}$ from $P(\boldsymbol{y})$ and then samples atomic configurations $\boldsymbol{x}$ from $R_{\boldsymbol{\phi }}(\boldsymbol{x} | \boldsymbol{y})$. Since $\boldsymbol{x}$ is discrete, the Gumbel softmax trick^30,31^ is applied. This technique uses Gumbel-distributed noise to approximate the sampling of $\boldsymbol{x}$ from $R_{\boldsymbol{\phi }}(\boldsymbol{x} | \boldsymbol{y})$ with a softmax function. This enables gradient-based optimization through the categorical sampling process, allowing backpropagation to train the model effectively.

Two neural networks are trained in parallel. The first plays the role of an encoder that predicts $\boldsymbol{\phi }$ parameters of $R_{\boldsymbol{\phi }}(\boldsymbol{x} | \boldsymbol{y})$, mapping auxiliary variables $\boldsymbol{y}$ to configurations $\boldsymbol{x}$. The second predicts the parameters $\boldsymbol{\theta }$ of $Q_{\boldsymbol{\theta }}(\boldsymbol{y}|\boldsymbol{x})$, in order to decode the sampled $\boldsymbol{x}$ back to $\boldsymbol{y}$. This is similar to the classical implementation of VAE^32^ but with inverse roles of encoder and decoder. Samples $\boldsymbol{y}$ from a traceable input distribution $P(\boldsymbol{y})$ are encoded into the space of atomic configurations approximated with $R_{\boldsymbol{\phi }}(\boldsymbol{x} | \boldsymbol{y})$ and then decoded back.

During each iteration of the training loop, where one batch of *M* variables $\boldsymbol{y}$ and configurations $\boldsymbol{x}$ is sampled, $-{\mathcal {L}}(\boldsymbol{\phi }, \boldsymbol{\theta })$ is estimated using the following formula:

6 $$\begin{aligned} -{\mathcal {L}}(\boldsymbol{\phi }, \boldsymbol{\theta }) \simeq \frac{1}{M}\sum _{i=1}^{M} \ln {\frac{f_T(\boldsymbol{x}^{(i)}) Q_{\boldsymbol{\theta }}(\boldsymbol{y}^{(i)}|\boldsymbol{x}^{(i)})}{ P(\boldsymbol{y}^{(i)}) R_{\boldsymbol{\phi }}(\boldsymbol{x}^{(i)} | \boldsymbol{y}^{(i)}) } }. \end{aligned}$$

*M* is one of the hyperparameters of the model. The sampling process can be customized in various ways; in this work, we sample one $\boldsymbol{x}$ for each sampled $\boldsymbol{y}$. The main computational limitation arises from the cost of energy minimization computations of $\boldsymbol{x}$ samples. While it is possible to sample multiple $\boldsymbol{x}$ per $\boldsymbol{y}$, in practice it is more efficient to sample a larger number of $\boldsymbol{y}$ values, each paired with a single $\boldsymbol{x}$, rather than sampling fewer $\boldsymbol{y}$ values with many $\boldsymbol{x}$ samples each. This allows for more frequent passes through the encoder and leads to faster model convergence.

### Experiments

 We apply PULSE to calculate the properties of bound Schottky defects (BSD) in a $\left(\mathrm{U},\mathrm{Pu}\right)\mathrm{O}_2$ MOX with varying Pu concentration. First, the machine learning model is tested and optimized by applying it to small atomic environments. These smaller systems allow for the computation of the true logarithm of the partition function, which can then be compared with the model predictions to assess accuracy. Next, the range of influence of atomic environments is measured to determine the appropriate size of the configurations to generate. Finally, after this initial testing and optimization, larger-scale experiments are conducted to obtain defect concentrations for different Pu concentrations and temperatures.

**Fig. 3:**
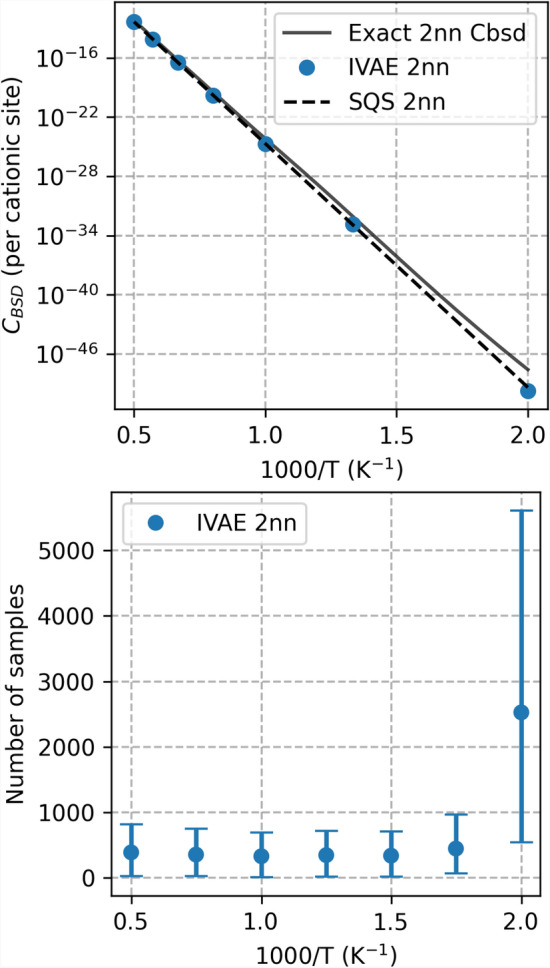
BSD3 defect concentration (left) and training cost (right) for the 2nn sphere of influence in a MOX with 50% Pu. Top panel: predictions generated by models achieving a minimum accuracy of 96.5%, determined by comparing the predicted logarithm of the partition function with analytically computed values using the 2nn BSD3 database developed in this study. The figure also includes SQS computations, presented as the average over $1280$ SQS configurations. The relative error and confidence interval for selected temperatures are provided in Table 1. Bottom panel: number of samples required to achieve 96.5% accuracy. Each estimate of the training cost is given as a 95% confidence interval derived from 40 models. The number of samples explicitly indicates the number of atomic configurations, and thus the required amount of CRG^33^ energy minimizations, that the model needed to generate during training to achieve at least 96.5% accuracy.

**Table 1:** Relative error between the $C_{\textrm{BSD}}$ values (Fig. 3), computed using the IVAE and SQS methods, and the exact result at selected temperatures. The relative error is calculated as: $\left|C_\mathrm{BSD(method)}-C_\mathrm{BSD(2nn\,exact)} \right|/C_\mathrm{BSD(2nn\,exact)}$. SQS estimates are shown as a 95% confidence interval based on $1280$ SQS supercell energy minimizations.

T [K]	IVAE	SQS
2000	0.26	$0.15 \pm 0.03$
1750	0.38	$0.19 \pm 0.04$
1500	0.45	$0.34 \pm 0.04$
1250	0.40	$0.35 \pm 0.04$
1000	0.56	$0.49 \pm 0.04$
750	0.84	$0.81 \pm 0.02$
500	0.98	$0.97 \pm 0.01$

In the following experiments, a simple neural network architecture with one hidden layer of $1024$ neurons and the Scaled Exponential Linear Unit (SELU) activation function was used for both the encoder $R_{\boldsymbol{\phi }}$ and decoder $Q_{\boldsymbol{\theta }}$ parts of the IVAE model. Reducing the hidden layer size below $1024$ neurons or adding additional layers did not lead to significant improvements in accuracy; however, both led to an increased training cost. The temperature parameter of the Gumbel softmax function was set to a fixed $\tau = 1/10$, and all experiments utilized the Adam optimizer with its default learning rate (0.001), and other default parameters as provided in TensorFlow 2.8. A Bernoulli distribution was used for $P(\boldsymbol{y})$. Various values of $|\boldsymbol{y}|$ and training batch sizes were tested, and $|\boldsymbol{y}| = 8$ with a training batch size of $M = 50$ atomic configurations per training step was selected, as this configuration yielded satisfactory results. All energy calculations were performed using the Cooper-Rushton-Grimes (CRG) potential^33^. All checkpoints during training of each model and the corresponding generated data were stored to ensure full traceability of each experiment. To provide a measure of the uncertainty, we employed a Deep Ensemble method by computing the standard deviation of the predictions across multiple models (from different random initializations) as a quantitative measure of the epistemic uncertainty associated with that estimate.

### Benchmarking machine learning models in the MOX system

In this section, the accuracy of the model predictions is evaluated. Calculating the exact partition function in a MOX environment is challenging; however, it becomes feasible in smaller environments due to the reduced configuration space. To assess the accuracy of the method, the predictions of the partition function are compared with numerically computed values from pre-prepared databases for first (1nn) and second (2nn) nearest neighbor (nn) spheres of influence. These databases include all possible configurations of U and Pu on the 12 (1nn) and 18 (2nn) atomic positions closest to the defect. A supercell was then constructed by adding atoms from a reference SQS supercell around each generated configuration. In this way, all configurations differed only in their local environments, while the surrounding external region remained identical for all cases. Subsequently, energy minimization was performed for each configuration, as well as on the same configurations containing a Schottky defect introduced at the center of the generated spheres, in order to compute the defect formation energies. The obtained energies were then used to compute the defect concentrations using Eq. (8), assuming that $w(\boldsymbol{x})$ is uniform for all configurations. This calculation served as the baseline for comparing the predictions of the IVAE models, and the result of the 2nn database is denoted as “Exact 2nn Cbsd” in later figures.

Tests were conducted for two sizes of the local environment and for three different Pu concentrations: 10%, 50%, and 90%. Fig. 3 shows the predictions of the PULSE approach for a MOX with a 50% Pu concentration in a 2nn system, along with the number of sampled atomic configurations required for the model to achieve a minimum accuracy of 96.5%. For comparison, the figure also includes SQS results, computed using Eq. (8) with constant $w(\boldsymbol{x}) = 1/N_{\textrm{SQS}}$ from $N_{\textrm{SQS}} = 1\,280$ SQS configurations. The numerical values and confidence intervals for selected temperatures are provided in Table 1. The SQS supercells are built using the same procedure applied for IVAE models, except that the 2nn local environment around the defect is taken from the generated SQS supercells.

These results demonstrate the capability of the PULSE approach to calculate partition functions that are nearly identical to those computed numerically, without significant computational overhead. In the 2nn system, the PULSE method required only to $6000$ configurations for training, compared to the configurations in the full 2nn database. The computational cost increases notably only at low temperatures. This issue arises from the difficulty of identifying the relevant states in the low-temperature regime, where a small number of states have a large impact on the partition function due to the Boltzmann factor. In practice, this means that the model needs to sample a larger number of configurations to provide a reliable estimate of the logarithm of the partition function. At high temperatures, assuming an approximate computational time of 5 CPU minutes per energy minimization of a atom supercell using the CRG potential^33^, the IVAE approach results in a total computational cost of 85 CPU hours, compared to $22282$ CPU hours if the entire 2nn database were processed. 

Additional evidence supporting the validity of the IVAE models is that their output is very close to the values obtained with SQS. The SQS results show a slightly better agreement with the analytical solution. This is expected, since SQS is well suited for ideally disordered solutions, which the MOX system approaches according to the CRG potential^33^ used for the energy minimization of the generated configurations, as discussed in earlier work^34^. The IVAE approach, however, also provides a clear criterion for assessing convergence, and can be easily adapted to non-ideal systems for which the SQS method is not as well suited.

### Measuring the range of influence of the local environment around the defect in the MOX system

**Fig. 4:**
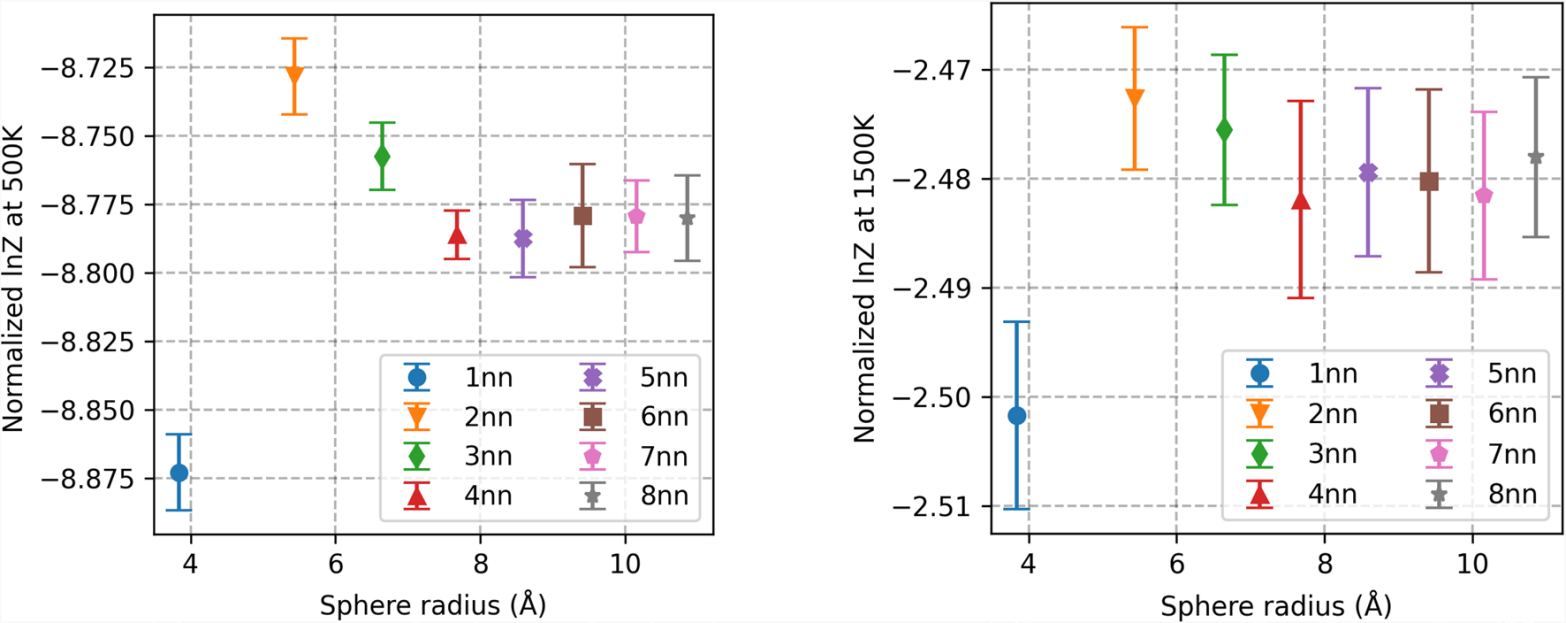
Range of influence of the local atomic environment around the defect, estimated with PULSE. Both graphs show the predictions of the model in a MOX system with a 50% Pu concentration for two different temperatures: 500 K on the left and $1500$ K on the right. The *y*-axis corresponds to the estimation of $\ln {(Z_T)}/n$, where $n = 12$. Error is reported as a 95% confidence interval based on 40 estimations of the partition function, reflecting epistemic uncertainty from data generated during training.

In this section, PULSE is employed to measure the defect range of influence in its local atomic environment. The IVAE model is configured to generate only the atoms closest to the defect (12 atoms in the 1nn sphere). Surrounding this inner sphere, U/Pu atoms are distributed randomly in shells from the 2nn to the 8nn distance, while all sites beyond the 8nn shell are filled according to a fixed U/Pu distribution from a reference supercell (see Section 4 for details). This setup ensures that the formation energy calculation is primarily determined by the IVAE-generated 1nn atoms, while the randomized 2nn–8nn shells allow assessment of how noise in the surrounding environment affects the IVAE 1nn generation, thereby defining the range of influence of the local atomic environment. The range of influence of local atomic environments was measured at two different temperatures, 500 K and $1500$ K, in the MOX system with a 50% Pu concentration. The results are shown in Fig. 4. As illustrated, the measured range of influence varies with temperature. At lower temperatures, the model predictions converge around the 4nn sphere of influence. However, as the temperature increases, convergence occurs sooner, closer to the 3nn sphere. This is because the average contribution of each shell to formation energy decreases as the distance from the defect increases. At lower temperatures, energy differences between shells are accentuated due to the Boltzmann factor $\frac{1}{k_{\textrm{B}}T}$. This results in energy gaps, such as those between the 3nn and 4nn shells, having a more pronounced effect at lower temperatures compared to higher temperatures. Consequently, to obtain accurate predictions of the partition function, it is necessary to extend the analysis to include more distant shells at lower temperatures to ensure convergence.

**Fig. 5:**
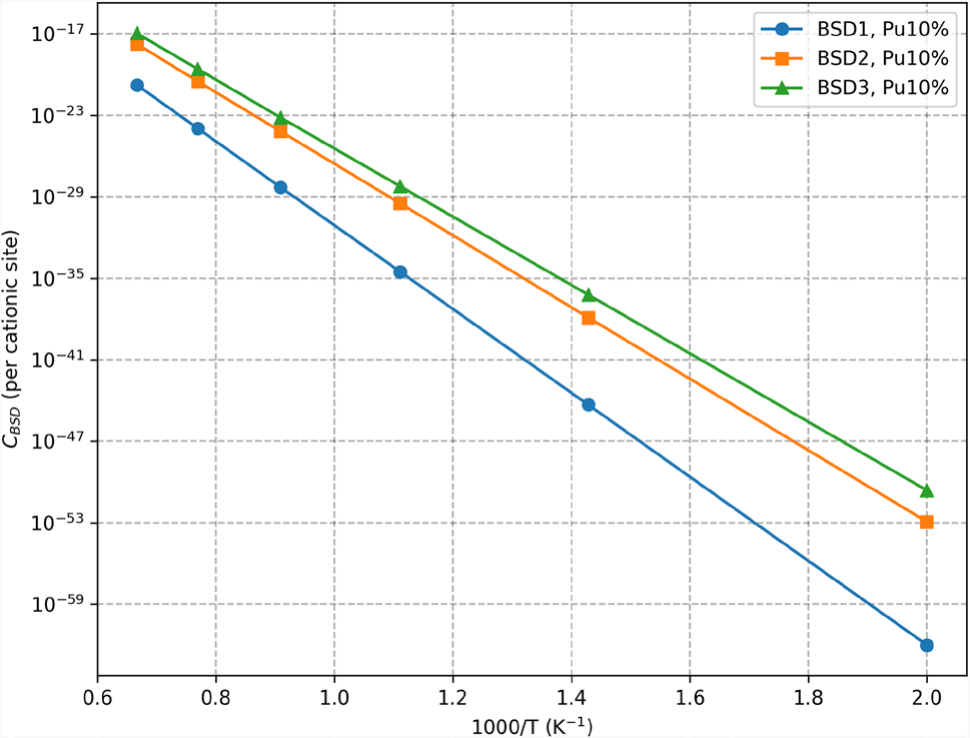
Comparison of the concentration $C_{\textrm{BSD}}(T)$ of different BSD defects (cf. Fig. 9) in $\mathrm {(U,Pu)O}_2$ with 10% Pu computed from 4nn predictions. Each point represents the mean of the predictions of the machine learning models. The details about the predictions and their variance are summarized in Table I in the Supplementary Information.

It is important to note that the model does not currently account for the entropic effects of finite temperature on the calculated formation energies. However, this limitation can be overcome by including finite-temperature effects in the atomic-scale calculations used to compute the defect formation energy, and the machine learning model can be adapted to incorporate this factor.

**Fig. 6:**
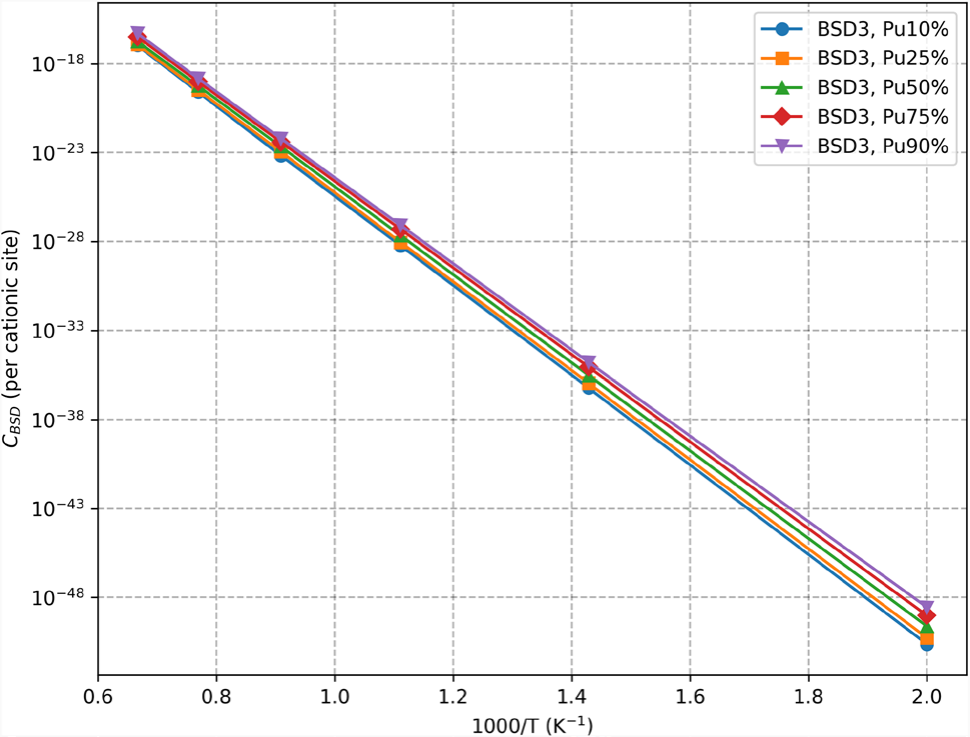
Comparison of the BSD3 (cf. Fig. 9) defect concentration $C_{\textrm{BSD}}(T)$ with varying Pu concentrations computed from 4nn predictions. Each point represents the mean of the predictions of the machine learning models. The details about the predictions and their variance are summarized in Table I in the Supplementary Information.

**Fig. 7:**
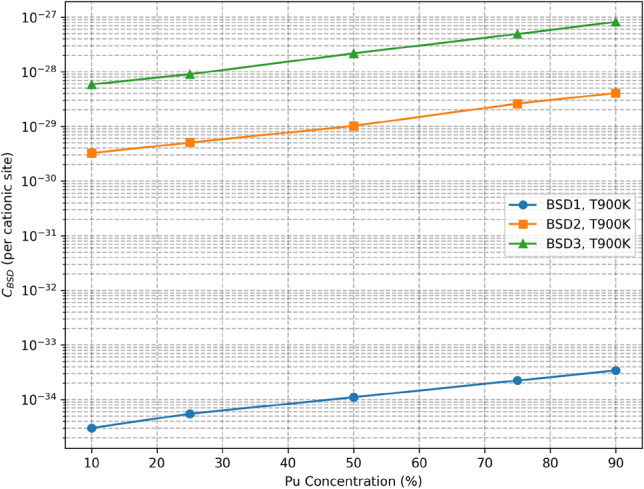
Comparison of the defect concentration $C_{\textrm{BSD}}(T)$ for different BSD types (cf. Fig. 9) at varying Pu concentrations in MOX at $T = 900$ K, computed from 4nn predictions. Each point represents the mean of the predictions of the machine learning models. The details about the predictions and their variance are summarized in Table I in the Supplementary Information.

**Fig. 8:**
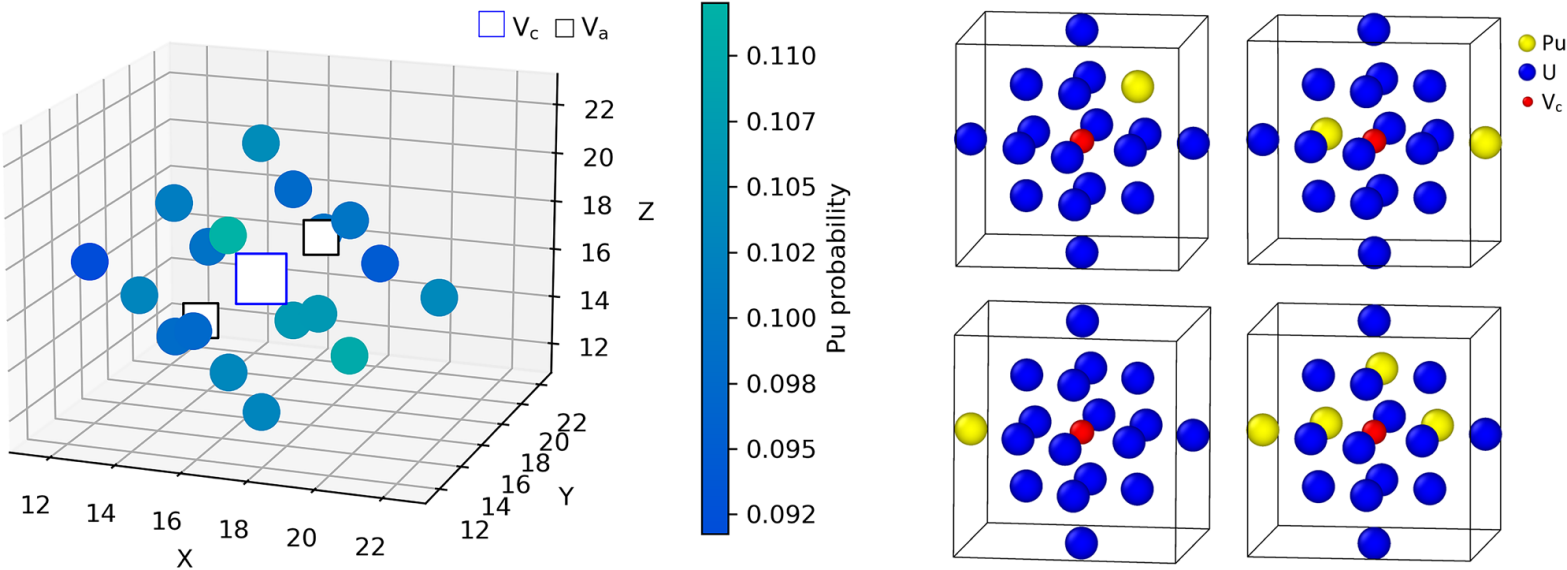
(left) 3D representation of the IVAE probability distribution, which represents the average occupancy on each atomic site surrounding the BSD3 defect for 2nn atomic configurations in MOX at $T = 500$ K, with a 10% Pu concentration. (right) Examples of 2nn configurations sampled from the multidimensional distribution $R_{\boldsymbol{\phi }}$. The 3D visualization of the IVAE probability distribution was computed by averaging probabilities for each cationic site predicted by $R_{\boldsymbol{\phi }}$. Oxygen and cation vacancies are marked as $\mathrm {V_a}$ and $\mathrm {V_\textrm{c}}$, respectively.

### Defect concentration for different Pu concentrations

PULSE is used to predict the defect concentration of three types of Schottky defects for five Pu concentrations: 10%, 25%, 50%, 75%, 90%, and temperatures ranging from 500 K to $1500$ K. We use all the results and optimizations we have found so far in the previous experiments. To ensure that the local environment around the defect is large enough, 4nn spheres are generated. We train all models on $2000$ atomic configurations to ensure accurate predictions, except for the models at $T = 500$ K, which are trained on $6000$ configurations.

Figure 5 presents predictions made for three types of defects at different temperatures and for 10% Pu concentration. Figure 6 compares different Pu concentrations for BSD3. The goal is to predict the highest value of its loss function, which serves as a lower bound of the logarithm of the true partition function. Therefore, the values in the aforementioned figures correspond to the mean of the 20 latest predictions from each model. Complete results, including exact calculations and computed standard deviations, are summarized in Table I in the Supplementary Information. Additionally, the effective formation energy of predicted defect concentrations is computed using the following formula:

7 $$\begin{aligned} E_{\textrm{eff}}^{\textrm{f}} = -k_{\textrm{B}}T\log {\left( C_\textrm{BSD}(T)\right) }. \end{aligned}$$

The computed effective energies are presented in the Supplementary Information.

The variations observed in Fig. 5 stem from differences in BSD stability predicted by the CRG potential. These results align with previous findings^34^. Additionally, Fig. 7 demonstrates an increase in defect concentration with higher Pu concentrations. This is consistent with a previous study^35^ showing that the formation energy of defects is generally lower in $\mathrm {PuO}_2$ compared to $\mathrm {UO}_2$, making it energetically more favorable for defects to form as Pu concentration increases. However, the increase in defect concentration with higher Pu concentration, while noticeable for lower temperatures, quickly diminishes with the temperature increase, as shown in Fig. 6.

To the authors’ knowledge, no experimental studies on BSD defect concentrations in MOX are currently available for comparison. PULSE is therefore a valuable tool for calculating properties that are difficult to access with other modeling methods, and sometimes challenging to measure experimentally. An interesting perspective for future work is to apply this approach to local environment-dependent properties in other multicomponent materials, such as HEAs, where experimental results are more readily obtainable.

### Visualizing IVAE predictions

The multidimensional probability distribution of configurations $R_{\boldsymbol{\phi }} (\boldsymbol{x} | \boldsymbol{y})$ can be visualized as an average occupancy, as shown in Fig. 8. Here, the probabilities of all components of $R_{\boldsymbol{\phi }} (\boldsymbol{x} | \boldsymbol{y})$ are averaged for each atomic site from 50 generated configurations, for which the calculated partition function was the highest. This allows for identifying physically relevant variations from the average Pu composition, which could help us to analyze the results in terms of, for instance, local ordering introduced by the presence of the point defect.

## Conclusions

This work demonstrates how PULSE can be applied to investigate local-atomic dependent properties in disordered compounds. It facilitates the estimation of partition functions and the sampling of configurations from complex configuration spaces at a limited computational cost. This approach offers a unique advantage, enabling targeted calculations of atomic-scale properties that would otherwise be extremely challenging to obtain using traditional methods, which are either too computationally expensive (e.g., Monte Carlo) or lead to incomplete sampling (e.g., SQS).

PULSE features an inverse variational autoencoder (IVAE) trained in an unsupervised manner, with no need for a preexisting training database, to provide accurate estimates of partition functions. The method was applied to estimate equilibrium defect concentrations and the range of influence of local atomic environments around the defect. Thanks to its versatility, the PULSE framework can be applied to a wide range of sampling tasks and systems, providing precise control over the sampling process. Overall, this method offers a significant advantage in generating representative configuration sets for target properties, which can be valuable, e.g., for constructing optimized training databases for interatomic potentials.

## Methods

### Concentration of thermal defects

The concentration of vacancy-type defects^34^ can be defined as:

8 $$\begin{aligned} C_{\textrm{BSD}}(T) = \sum _{\boldsymbol{x} \in \boldsymbol{X}} w(\boldsymbol{x}) \exp \left( {-\frac{E_{\textrm{BSD}}^{\textrm{f}}(\boldsymbol{x})}{k_{\textrm{B}}T}}\right) . \end{aligned}$$

**Fig. 9:**
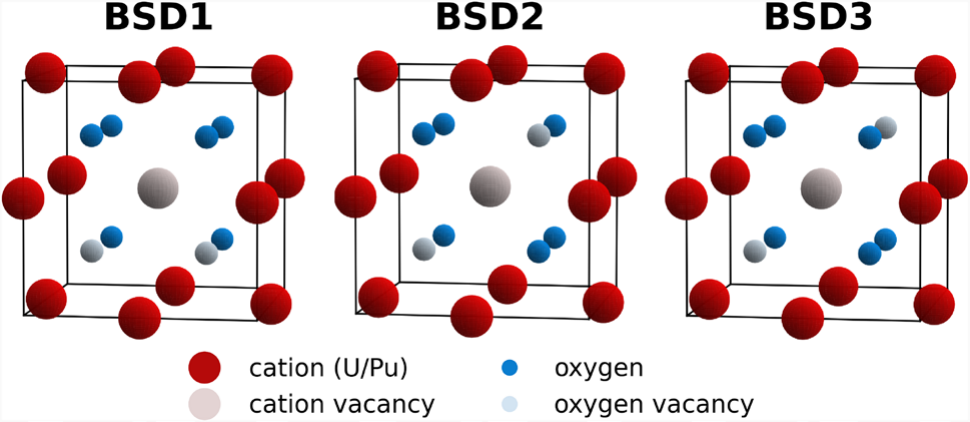
Illustration of three distinct bound Schottky defects in the fluorite structure of $\mathrm {(U,Pu)O}_2$ (color figure online).

$\boldsymbol{x}$ corresponds to atomic configurations $\boldsymbol{x} \in \boldsymbol{X}$; namely, a distribution of U/Pu atoms on the cationic sublattice, where $\boldsymbol{X} \in {\mathbb {R}}^{3N}$ represents the configuration space defined by the atomic position coordinates. Specifically, this space $\boldsymbol{X}$ consists of all possible $\boldsymbol{x}$ where all degenerate symmetrically equivalent configurations are counted individually, ensuring that the multiplicity of each configuration is strictly equal to 1. A cation and two oxygen atoms must be removed from the supercell to form a bound Schottky defect (BSD). In all experiments, defects were created by removing a U atom and two oxygen atoms, similar to a previous study^34^. The various BSD studied in this work are presented in Fig. 9. $E_{\textrm{BSD}}^{\textrm{f}}(\boldsymbol{x})$ denotes the formation energy of a vacancy-type Schottky defect, computed with Eq. (13), and the sum in Eq. (8) extends over all $\boldsymbol{x}$ in $\boldsymbol{X}$.

Here, $w(\boldsymbol{x})$ is the normalized weight corresponding to the probability of configuration $\boldsymbol{x}$. For simplicity, we assume in this work that $w(\boldsymbol{x})$ can be approximated by the configuration probability in ideal solid solutions. This approximation will be removed in future work. In ideal solid solutions, the probability $p(n_{\textrm{Pu}})$ of having a given number of Pu atoms in an atomic configuration (representing the local environment around the defect) can be described using the binomial distribution formula:

9 $$\begin{aligned} p(n_{\textrm{Pu}})= \left( {\begin{array}{c}M_\textrm{C}\\ n_{\textrm{Pu}}\end{array}}\right) \hspace{2pt} y_{\textrm{Pu}}^{n_{\textrm{Pu}}} \hspace{2pt} (1 - y_{\textrm{Pu}})^{n_{\textrm{U}}}. \end{aligned}$$

$M_\textrm{C}$ is the number of cations in the considered configuration, while $n_{\textrm{Pu}}$ and $n_{\textrm{U}}$ represent the number of Pu and U respectively, so that $M_\textrm{C} = n_{\textrm{U}} + n_{\textrm{Pu}}$. Pu concentration is expressed with $y_{\textrm{Pu}}$, so that $y_{\textrm{U}} = 1 - y_{\textrm{Pu}}$. From Eq. (9) we define $w(\boldsymbol{x})$ as:

10 $$\begin{aligned} w(\boldsymbol{x}) = y_{\textrm{Pu}}^{n_{\textrm{Pu}}^{\boldsymbol{x}}} \hspace{2pt} (1 - y_{\textrm{Pu}})^{ n_{\textrm{U}}^{\boldsymbol{x}}}. \end{aligned}$$

In Eq. (10), $n_\textrm{Pu}^{\boldsymbol{x}}$ ($n_\textrm{U}^{\boldsymbol{x}}$) denote the number of Pu (U) atoms within the atomic configuration $\boldsymbol{x}$, so that $M_\textrm{C} = n_\textrm{Pu}^{\boldsymbol{x}} + n_\textrm{U}^{\boldsymbol{x}}$. It can be shown that if we consider all $\boldsymbol{x} \in \boldsymbol{X}$, then $\sum _{\boldsymbol{x} \in \boldsymbol{X}} w(n_\textrm{Pu}^{\boldsymbol{x}}) = 1$. Removing $$\left( {\begin{array}{c}M_\textrm{C}\\ n_{\textrm{Pu}}\end{array}}\right)$$ is necessary, because instead of iterating through all $n_\textrm{Pu} \in [0, M_\textrm{C}]$, as in Eq. (9), in Eq. (8) we iterate through all $\boldsymbol{x} \in \boldsymbol{X}$.

We denote the exponential term in Eq. (8) as $f_T(\boldsymbol{x})$, so that for a given temperature $T$:

11 $$\begin{aligned} f_T(\boldsymbol{x}) = \exp \left( {-\frac{E_{\textrm{BSD}}^{\textrm{f}}(\boldsymbol{x})}{k_{\textrm{B}}T}} + \ln {\left( w(\boldsymbol{x})\right) } \right) , \end{aligned}$$

and

12 $$\begin{aligned} C_{\textrm{BSD}}(T) = \sum _{\boldsymbol{x} \in \boldsymbol{X}} f_T(\boldsymbol{x}) = Z_T. \end{aligned}$$

$Z_T$ is referred to as the partition function associated with the formation energy. Therefore, by estimating $Z_T$, one can directly obtain the defect concentration $C_{\textrm{BSD}}(T)$. The term “partition function” is used here for $Z_T$ to facilitate the discussion and demonstrate the general applicability of the PULSE approach in evaluating partition functions of this form. However, it is important to remind that $Z_T$ does not correspond to the actual partition function describing the thermodynamic equilibrium of the system. Nevertheless, the methodology presented here can, in principle, be applied to any type of partition function.

### Description of the atomic environment in the MOX system

In our application, generating configurations involves two steps. First, configurations are generated with atoms in the closest vicinity of the BSD, called nearest neighbor (nn) spheres of configurations or spheres of influence. This reduces the configuration space to possible atomic configurations on the $x$nn sphere, where $x$ varies depending on the sphere radius. Although this space reduction is an approximation, as explained in Sec. 2, it is sufficient to provide satisfactory results on point defect properties, while lowering the computational cost. Second, the environment surrounding the generated spheres is constructed, and periodic boundary conditions are applied to the full supercell. To prevent interactions between the defect and its periodic images, the size of the supercell is set to $2592$ atoms (864 cations and $1728$ anions). This represents a $6 \times 6 \times 6$ replication of a primitive cell of a fluorite structure.

The environment surrounding each generated sphere is prepared in two ways. The first method uses a reference supercell, where its central atoms are replaced with those generated by the IVAE model, following the procedure described in a previous work^34^. This keeps the surrounding environment unchanged across experiments, but may introduce slight variations in the total Pu concentration since the IVAE model (in its current implementation) is not constrained to place an exact number of Pu atoms within the generated configurations. The second approach involves randomly filling the environment around nn spheres to achieve the desired Pu concentration. For each generated sphere, the number of placed Pu atoms is counted, and the rest of the supercell is randomly filled to reach the target total number of Pu atoms in the supercell. The first method (reference supercell) is used for studying the range of influence and benchmarking the machine learning models, while the remaining experiments use the second method (random initialization of the surrounding environment).

In the first approach, it should be noted that a fluctuating Pu concentration may bias the estimation of defect concentrations. Nevertheless, given the large size of the supercells, fluctuations among the generated supercells are minimal (a few percent at most) and can be safely considered negligible in terms of their effect on defect formation energy.

### Energy minimization

The BSD formation energies in each configuration are obtained via classical molecular statics (i.e., 0-K energy minimization) using the LAMMPS code^36^ and the Cooper-Rushton-Grimes (CRG) interatomic potential for actinide oxides^33^. As a reminder, the formation energy for each configuration involves energy minimization of two supercells: one with and one without a defect. All supercells with a defect were prepared by removing a U atom and two O atoms to form a BSD, and the formation energy was calculated as^34^:

13 $$\begin{aligned} E_{\textrm{BSD}}^{\textrm{f}}(\boldsymbol{x})&=E_{\textrm{BSD}}(\boldsymbol{x}) - E(\boldsymbol{x}) + \frac{E(\mathrm {UO}_2)}{N_\textrm{C}}. \end{aligned}$$

$E(\mathrm {UO}_2)$ is the energy of the pure $\mathrm {UO}_2$ supercell containing $N_\textrm{C}$ cations.

An alternative approach to computing the formation energy is to remove one Pu atom and two O atoms, and use $E(\mathrm {PuO}_2)$ as the energy of the pure $\mathrm {PuO}_2$ supercell instead of $E(\mathrm {UO}_2)$ in Eq. (13). As discussed in a previous work^34^, the two approaches are strictly equivalent for ideal solid solutions, whereas they differ in the presence of local-order effects. For $\mathrm {(U,Pu)O}_2$ oxides, this effect is minor, at least according to the CRG potential^34^. Additional evidence for the absence of strong local-order effects is given by the Warren–Cowley short-range order parameter, reported in Fig. 4 of the Supplementary Information, which gradually converges to zero during IVAE training for all nn shells. For this reason, all formation energy calculations in this paper are performed using Eq. (13) with the energy of the pure $\mathrm {UO}_2$ supercell $E(\mathrm {UO}_2)$.

In Eqs. (8) and (13), all finite temperature contributions to the defect formation energy are omitted. However, these contributions can be straightforwardly integrated by replacing the formation enthalpy $E_\textrm{BSD}^\textrm{f}$ with the defect free formation energy in the same equations, while the working principles of the IVAE remain entirely unchanged. Such contributions may be evaluated using harmonic approximations or thermodynamic integration techniques within molecular dynamics frameworks such as LAMMPS. While these effects are not explicitly considered in the present work, the approach developed here can be readily extended to include them, offering a pathway toward a more comprehensive thermodynamic description in future applications.

### Indicator function

For the specific case of $\mathrm {(U,Pu)O}_2$, each element $x^{(i)}$ of the configuration $\boldsymbol{x}$ corresponds to either Pu or U. In Eq. (3), an indicator function $I(x^{(i)})$ is introduced to assign values 1 or −1 to Pu or U elements of $\boldsymbol{x}$, respectively:

14 $$\begin{aligned} I(x^{(i)}) = {\left\{ \begin{array}{ll} \phantom {-}1 & \text {if } x^{(i)} \text { represents Pu,} \\ -1 & \text {if } x^{(i)} \text { represents U.} \end{array}\right. } \end{aligned}$$

## Supplementary Information

Supplementary Information.

## Data Availability

The data underlying the findings of this study are available in the Supplementary Information or from the corresponding author upon request.
