# Supplementary material for: AI-driven data-efficient estimation of partition functions in disordered materials
Source: Sci Rep. 2026 Mar 23;16:14568. doi: 10.1038/s41598-026-37953-6 (PMC13153157; doi:10.1038/s41598-026-37953-6)
Supplement: Supplementary file 1 — Supplementary Information. [file 41598_2026_37953_MOESM1_ESM.pdf]

Supplementary Information for "AI-driven data-efficient estimation of partition functions in disordered materials"

Maciej J. Karcz[1,2], Luca Messina[1], Eiji Kawasaki[2], Emeric Bourasseau[1]
[1] *CEA, DES, IRESNE, DEC, Cadarache, F-13108, Saint-Paul-Lez-Durance, France*
[2] *Université Paris-Saclay, CEA, LIST, F-91120, Palaiseau, France*

CONTENTS

This document presents additional tables and figures, including calculations and associated standard deviations.

Tables I and II report the bound Schottky defect (BSD) concentrations in $(U,Pu)O_2$ mixed oxides (MOX) and the corresponding effective formation energies (Eq. 7 in the main paper), visualized in Figs. 1, 2, and 3 as functions of Pu concentration and temperature. Overall, the effective BSD formation energy decreases with both increasing Pu concentration and temperature.

Figure 4 shows the evolution of the Warren–Cowley (WC) parameter during training for configurations generated by one IVAE model. The WC parameter $\alpha^{x\mathrm{nn}}_{\mathrm{U-Pu}}$ is computed as:

$$\alpha^{x\mathrm{nn}}_{\mathrm{U-Pu}} = 1 - \frac{p^{x\mathrm{nn}}_{\mathrm{U-Pu}}}{c_{\mathrm{Pu}}} \, , \tag{1}$$

for U–Pu nearest-neighbor (nn) shells from 1nn to 4nn. Here, $p^{x\mathrm{nn}}_{\mathrm{U-Pu}}$ is the probability of finding a Pu atom on the $x$nn shell around a U atom, and c_{Pu} is the nominal Pu concentration. The results indicate that, during training, the parameter approaches zero across all shells, demonstrating that the PULSE method converges toward a nearly random distribution of Pu atoms.

Finally, Figure 5 presents IVAE experiments on the square-lattice Ising model, consisting of binary spins that mimic two-species occupancy in a lattice, used here as a proxy for binary compounds. In these tests, we considered the standard nearest-neighbor Ising setup with spins x_i and Hamiltonian $H(\mathbf{x})$:

$$H(\mathbf{x}) = -J \sum_{(i,j)} x_i x_j - h \sum_i x_i \, , \tag{2}$$

with coupling $J = 1$ and no external field ($h = 0$). The IVAE architecture matches that used in the main paper, i.e., a single hidden layer with 1024 units, SELU activation, Gumbel–Softmax relaxation with $\tau = 0.1$, and the Adam optimizer with the default parameters of TensorFlow v2.8. The results show that IVAE accurately estimates the partition function across all tested system sizes, and that the number of samples required to reach 99% accuracy remains roughly constant.

TABLE I. Concentration of bound Schottky defects (BSD) $C_{\mathrm{BSD}}(T)$ per cationic site for Pu concentrations ranging from 10% to 90% and temperatures from 500 to 1 500 K. The types of BSDx correspond to the schematics in Fig. 9 of the main paper. Each prediction is given by a separate IVAE model, i.e., 90 models (5 Pu concentrations, 6 temperatures, and 3 defect types) were applied in total. All models, except those for $T = 500$ K, were tasked to generate 2 000 4nn configurations during training, 50 configurations per training step, with $|\mathbf{y}| = 8$. Models for $T = 500$ K were tasked to generate 6 000 configurations. The computed values of defect concentrations are given as a mean ±2 standard deviations, calculated from the 20 latest predictions of the IVAE models.

Environment	BSD1	BSD2	BSD3
$y_{\mathrm{Pu}} = 10\%$, 500 K	$(9.71 \pm 4.86) \times 10^{-63}$	$(1.14 \pm 0.68) \times 10^{-53}$	$(2.37 \pm 1.45) \times 10^{-51}$
$y_{\mathrm{Pu}} = 10\%$, 700 K	$(4.76 \pm 2.23) \times 10^{-45}$	$(1.26 \pm 0.53) \times 10^{-38}$	$(5.99 \pm 3.20) \times 10^{-37}$
$y_{\mathrm{Pu}} = 10\%$, 900 K	$(3.01 \pm 1.18) \times 10^{-35}$	$(3.25 \pm 0.92) \times 10^{-30}$	$(5.89 \pm 2.38) \times 10^{-29}$
$y_{\mathrm{Pu}} = 10\%$, 1 100 K	$(5.02 \pm 2.04) \times 10^{-29}$	$(6.66 \pm 2.26) \times 10^{-25}$	$(6.85 \pm 2.50) \times 10^{-24}$
$y_{\mathrm{Pu}} = 10\%$, 1 300 K	$(1.10 \pm 0.32) \times 10^{-24}$	$(3.18 \pm 1.25) \times 10^{-21}$	$(2.59 \pm 0.82) \times 10^{-20}$
$y_{\mathrm{Pu}} = 10\%$, 1 500 K	$(1.72 \pm 0.85) \times 10^{-21}$	$(1.82 \pm 0.59) \times 10^{-18}$	$(1.08 \pm 0.32) \times 10^{-17}$
$y_{\mathrm{Pu}} = 25\%$, 500 K	$(3.48 \pm 3.11) \times 10^{-62}$	$(2.72 \pm 1.58) \times 10^{-53}$	$(5.31 \pm 4.64) \times 10^{-51}$
$y_{\mathrm{Pu}} = 25\%$, 700 K	$(7.98 \pm 6.24) \times 10^{-45}$	$(2.30 \pm 1.33) \times 10^{-38}$	$(1.10 \pm 0.55) \times 10^{-36}$
$y_{\mathrm{Pu}} = 25\%$, 900 K	$(5.48 \pm 4.03) \times 10^{-35}$	$(5.07 \pm 2.46) \times 10^{-30}$	$(9.05 \pm 5.37) \times 10^{-29}$
$y_{\mathrm{Pu}} = 25\%$, 1 100 K	$(6.66 \pm 2.78) \times 10^{-29}$	$(8.60 \pm 4.48) \times 10^{-25}$	$(1.13 \pm 0.42) \times 10^{-23}$
$y_{\mathrm{Pu}} = 25\%$, 1 300 K	$(1.41 \pm 0.55) \times 10^{-24}$	$(4.72 \pm 2.03) \times 10^{-21}$	$(3.39 \pm 1.77) \times 10^{-20}$
$y_{\mathrm{Pu}} = 25\%$, 1 500 K	$(1.97 \pm 0.90) \times 10^{-21}$	$(1.95 \pm 0.92) \times 10^{-18}$	$(1.24 \pm 0.72) \times 10^{-17}$
$y_{\mathrm{Pu}} = 50\%$, 500 K	$(1.38 \pm 1.66) \times 10^{-61}$	$(1.45 \pm 1.18) \times 10^{-52}$	$(2.40 \pm 3.10) \times 10^{-50}$
$y_{\mathrm{Pu}} = 50\%$, 700 K	$(2.43 \pm 1.62) \times 10^{-44}$	$(8.39 \pm 6.99) \times 10^{-38}$	$(3.01 \pm 1.97) \times 10^{-36}$
$y_{\mathrm{Pu}} = 50\%$, 900 K	$(1.10 \pm 0.75) \times 10^{-34}$	$(1.02 \pm 0.69) \times 10^{-29}$	$(2.18 \pm 1.46) \times 10^{-28}$
$y_{\mathrm{Pu}} = 50\%$, 1 100 K	$(1.20 \pm 0.50) \times 10^{-28}$	$(1.91 \pm 0.90) \times 10^{-24}$	$(2.19 \pm 1.19) \times 10^{-23}$
$y_{\mathrm{Pu}} = 50\%$, 1 300 K	$(2.42 \pm 1.11) \times 10^{-24}$	$(7.19 \pm 3.60) \times 10^{-21}$	$(5.71 \pm 2.86) \times 10^{-20}$
$y_{\mathrm{Pu}} = 50\%$, 1 500 K	$(3.14 \pm 1.95) \times 10^{-21}$	$(3.13 \pm 1.60) \times 10^{-18}$	$(1.83 \pm 0.80) \times 10^{-17}$
$y_{\mathrm{Pu}} = 75\%$, 500 K	$(3.65 \pm 4.11) \times 10^{-61}$	$(5.89 \pm 5.48) \times 10^{-52}$	$(9.57 \pm 5.89) \times 10^{-50}$
$y_{\mathrm{Pu}} = 75\%$, 700 K	$(6.36 \pm 3.35) \times 10^{-44}$	$(2.01 \pm 1.15) \times 10^{-37}$	$(8.79 \pm 7.58) \times 10^{-36}$
$y_{\mathrm{Pu}} = 75\%$, 900 K	$(2.23 \pm 1.41) \times 10^{-34}$	$(2.62 \pm 1.38) \times 10^{-29}$	$(4.93 \pm 2.14) \times 10^{-28}$
$y_{\mathrm{Pu}} = 75\%$, 1 100 K	$(2.69 \pm 1.44) \times 10^{-28}$	$(3.69 \pm 1.93) \times 10^{-24}$	$(3.97 \pm 2.03) \times 10^{-23}$
$y_{\mathrm{Pu}} = 75\%$, 1 300 K	$(4.02 \pm 1.84) \times 10^{-24}$	$(1.28 \pm 0.59) \times 10^{-20}$	$(9.77 \pm 3.93) \times 10^{-20}$
$y_{\mathrm{Pu}} = 75\%$, 1 500 K	$(5.07 \pm 2.55) \times 10^{-21}$	$(5.83 \pm 2.54) \times 10^{-18}$	$(3.27 \pm 1.76) \times 10^{-17}$
$y_{\mathrm{Pu}} = 90\%$, 500 K	$(9.47 \pm 6.69) \times 10^{-61}$	$(1.47 \pm 0.86) \times 10^{-51}$	$(2.80 \pm 1.95) \times 10^{-49}$
$y_{\mathrm{Pu}} = 90\%$, 700 K	$(1.14 \pm 0.49) \times 10^{-43}$	$(4.29 \pm 1.28) \times 10^{-37}$	$(1.63 \pm 0.74) \times 10^{-35}$
$y_{\mathrm{Pu}} = 90\%$, 900 K	$(3.40 \pm 1.12) \times 10^{-34}$	$(4.09 \pm 2.26) \times 10^{-29}$	$(8.20 \pm 3.22) \times 10^{-28}$
$y_{\mathrm{Pu}} = 90\%$, 1 100 K	$(3.61 \pm 1.21) \times 10^{-28}$	$(6.27 \pm 1.77) \times 10^{-24}$	$(6.23 \pm 2.09) \times 10^{-23}$
$y_{\mathrm{Pu}} = 90\%$, 1 300 K	$(5.93 \pm 1.83) \times 10^{-24}$	$(2.07 \pm 0.78) \times 10^{-20}$	$(1.50 \pm 0.56) \times 10^{-19}$
$y_{\mathrm{Pu}} = 90\%$, 1 500 K	$(7.22 \pm 1.74) \times 10^{-21}$	$(8.04 \pm 2.74) \times 10^{-18}$	$(5.18 \pm 1.84) \times 10^{-17}$

TABLE II. Effective formation energy, $E^{\mathrm{f}}_{\mathrm{eff}}$ [eV] (cf. Eq. 7 in the main paper), for Pu concentrations ranging from 10% to 90% and temperatures from 500 to 1 500 K, and three BSD types (cf. schematics in Fig. 9 of the main paper). Each effective energy was computed from the IVAE defect concentration in Table I. All models, except those for $T = 500$ K, were tasked to generate 2 000 4nn configurations during training, 50 configurations per training step, with $|\mathbf{y}| = 8$. Models for $T = 500$ K were tasked to generate 6 000 configurations. The computed values of effective formation energies are given as a mean ± 2 standard deviations, calculated from the 20 latest predictions of the IVAE models.

Environment	BSD1 [eV]	BSD2 [eV]	BSD3 [eV]
$y_{\mathrm{Pu}} = 10\%$, 500 K	6.15 ± 0.02	5.25 ± 0.02	5.02 ± 0.03
$y_{\mathrm{Pu}} = 10\%$, 700 K	6.16 ± 0.03	5.27 ± 0.03	5.03 ± 0.03
$y_{\mathrm{Pu}} = 10\%$, 900 K	6.17 ± 0.03	5.27 ± 0.02	5.04 ± 0.03
$y_{\mathrm{Pu}} = 10\%$, 1 100 K	6.18 ± 0.04	5.28 ± 0.03	5.06 ± 0.03
$y_{\mathrm{Pu}} = 10\%$, 1 300 K	6.18 ± 0.03	5.29 ± 0.04	5.05 ± 0.04
$y_{\mathrm{Pu}} = 10\%$, 1 500 K	6.18 ± 0.07	5.28 ± 0.04	5.05 ± 0.04
$y_{\mathrm{Pu}} = 25\%$, 500 K	6.10 ± 0.04	5.22 ± 0.03	4.99 ± 0.04
$y_{\mathrm{Pu}} = 25\%$, 700 K	6.13 ± 0.04	5.23 ± 0.03	5.00 ± 0.03
$y_{\mathrm{Pu}} = 25\%$, 900 K	6.12 ± 0.06	5.23 ± 0.03	5.01 ± 0.04
$y_{\mathrm{Pu}} = 25\%$, 1 100 K	6.15 ± 0.04	5.26 ± 0.05	5.01 ± 0.04
$y_{\mathrm{Pu}} = 25\%$, 1 300 K	6.15 ± 0.04	5.25 ± 0.05	5.03 ± 0.06
$y_{\mathrm{Pu}} = 25\%$, 1 500 K	6.17 ± 0.06	5.27 ± 0.06	5.04 ± 0.07
$y_{\mathrm{Pu}} = 50\%$, 500 K	6.05 ± 0.05	5.15 ± 0.04	4.93 ± 0.05
$y_{\mathrm{Pu}} = 50\%$, 700 K	6.06 ± 0.04	5.15 ± 0.04	4.94 ± 0.04
$y_{\mathrm{Pu}} = 50\%$, 900 K	6.07 ± 0.05	5.18 ± 0.05	4.94 ± 0.06
$y_{\mathrm{Pu}} = 50\%$, 1 100 K	6.10 ± 0.04	5.18 ± 0.05	4.95 ± 0.05
$y_{\mathrm{Pu}} = 50\%$, 1 300 K	6.09 ± 0.06	5.20 ± 0.06	4.97 ± 0.05
$y_{\mathrm{Pu}} = 50\%$, 1 500 K	6.11 ± 0.08	5.21 ± 0.07	4.98 ± 0.06
$y_{\mathrm{Pu}} = 75\%$, 500 K	6.00 ± 0.04	5.09 ± 0.04	4.87 ± 0.03
$y_{\mathrm{Pu}} = 75\%$, 700 K	6.00 ± 0.03	5.10 ± 0.03	4.87 ± 0.05
$y_{\mathrm{Pu}} = 75\%$, 900 K	6.01 ± 0.05	5.11 ± 0.04	4.88 ± 0.04
$y_{\mathrm{Pu}} = 75\%$, 1 100 K	6.02 ± 0.05	5.12 ± 0.05	4.89 ± 0.04
$y_{\mathrm{Pu}} = 75\%$, 1 300 K	6.04 ± 0.05	5.13 ± 0.05	4.91 ± 0.04
$y_{\mathrm{Pu}} = 75\%$, 1 500 K	6.04 ± 0.07	5.13 ± 0.06	4.91 ± 0.07
$y_{\mathrm{Pu}} = 90\%$, 500 K	5.96 ± 0.03	5.04 ± 0.02	4.82 ± 0.03
$y_{\mathrm{Pu}} = 90\%$, 700 K	5.97 ± 0.02	5.05 ± 0.02	4.83 ± 0.03
$y_{\mathrm{Pu}} = 90\%$, 900 K	5.98 ± 0.03	5.07 ± 0.04	4.84 ± 0.03
$y_{\mathrm{Pu}} = 90\%$, 1 100 K	5.99 ± 0.03	5.07 ± 0.03	4.85 ± 0.03
$y_{\mathrm{Pu}} = 90\%$, 1 300 K	5.99 ± 0.03	5.08 ± 0.04	4.86 ± 0.04
$y_{\mathrm{Pu}} = 90\%$, 1 500 K	6.00 ± 0.03	5.09 ± 0.04	4.85 ± 0.05

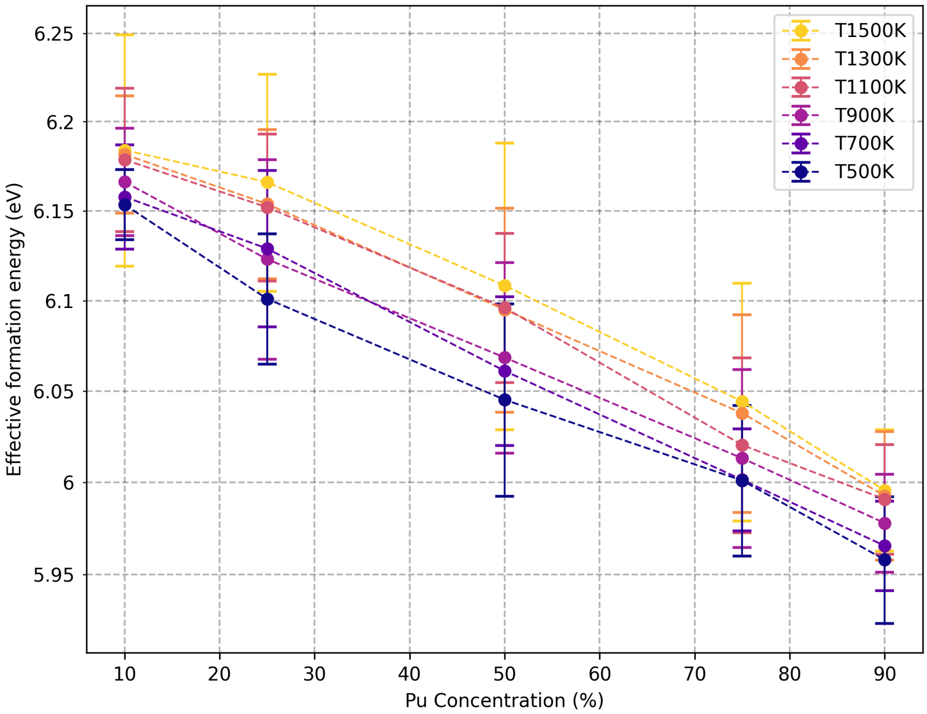

FIG. 1. BSD1 effective formation energy $E^{\mathrm{f}}_{\mathrm{eff}}$ in $(\mathrm{U,Pu})\mathrm{O}_2$ as a function of temperature and Pu concentration. The details about the predictions and the associated variance are summarized in Table II.

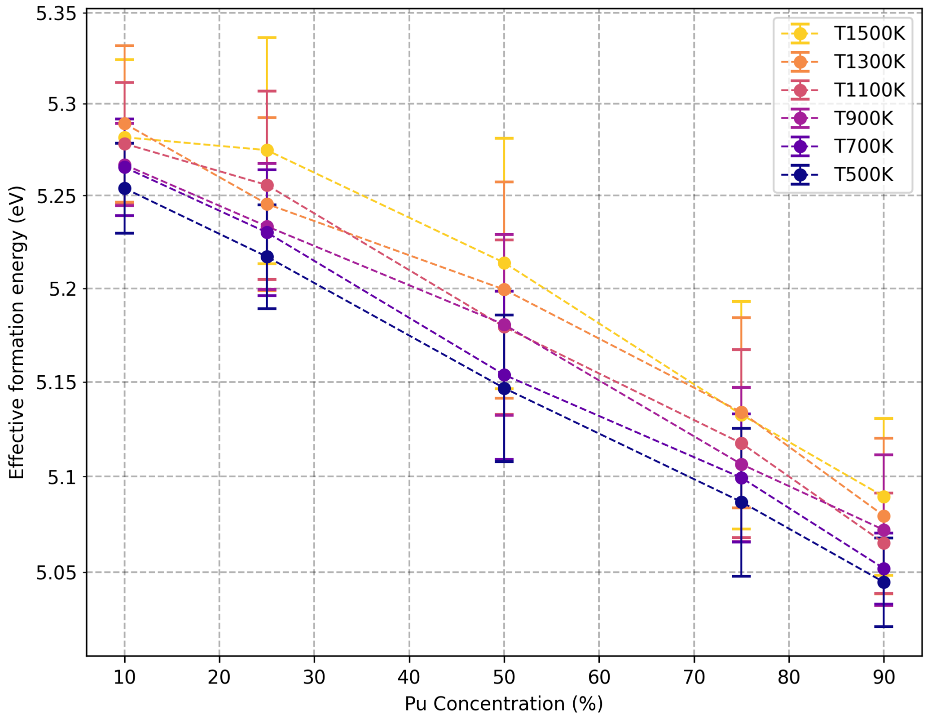

FIG. 2. BSD2 effective formation energy $E^{\mathrm{f}}_{\mathrm{eff}}$ in $(\mathrm{U,Pu})\mathrm{O}_2$ as a function of temperature and Pu concentration. The details about the predictions and the associated variance are summarized in Table II.

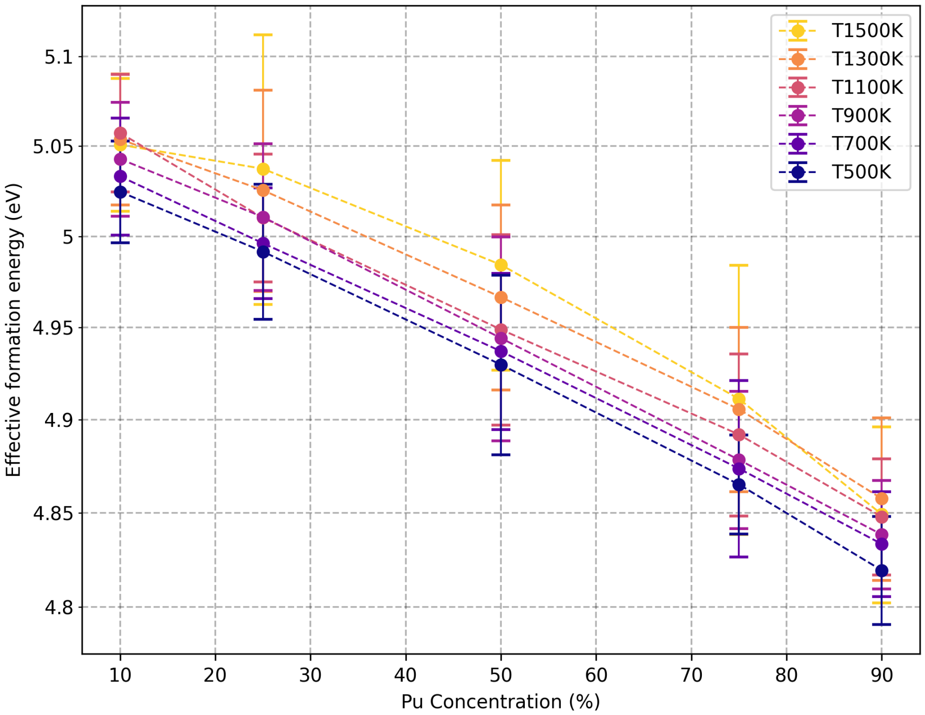

FIG. 3. BSD3 effective formation energy $E^{\mathrm{f}}_{\mathrm{eff}}$ in $(\mathrm{U},\mathrm{Pu})\mathrm{O}_2$ as a function of temperature and Pu concentration. The details about the predictions and the associated variance are summarized in Table II.

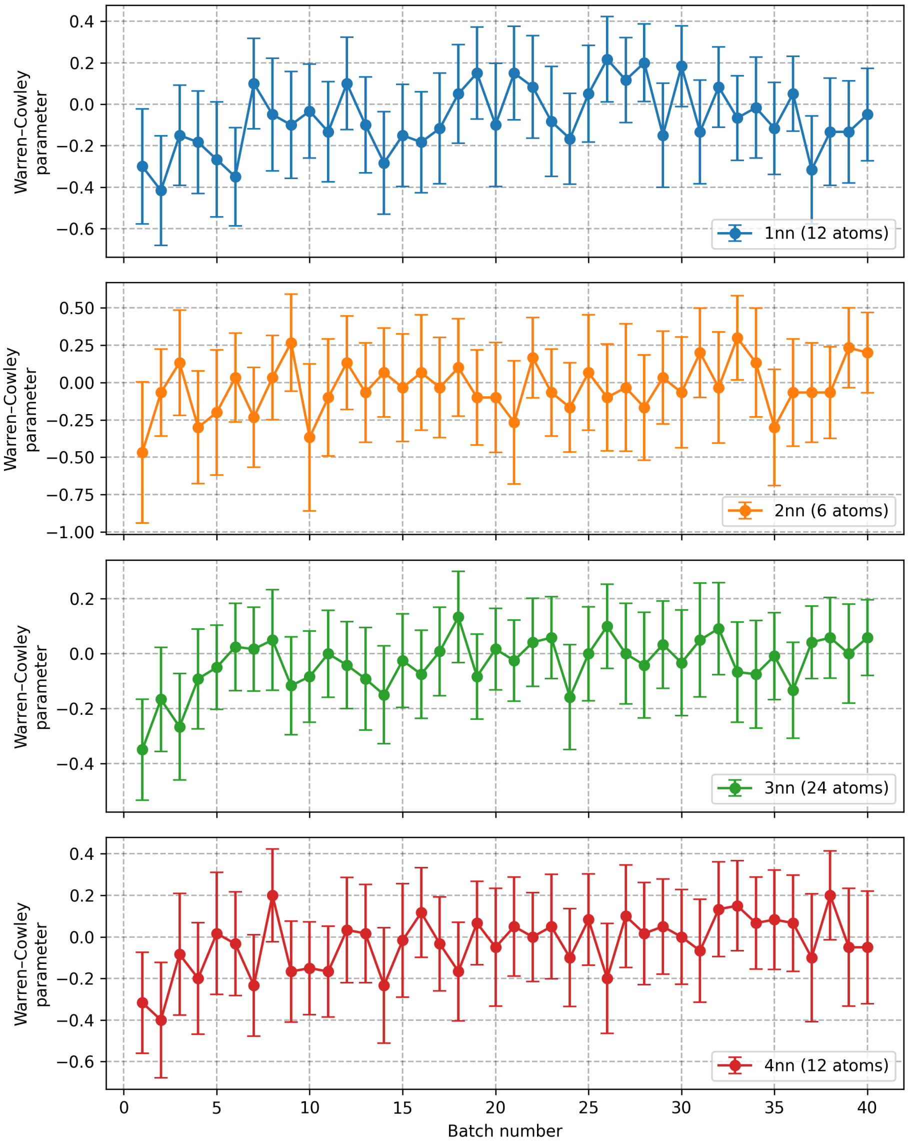

FIG. 4. Calculation of the Warren–Cowley parameter for different atomic shells generated by one of the IVAE models. The model was trained to generate 4nn configurations around a BSD3, with $T = 1\,100$ K and 10% Pu concentration. The Warren–Cowley parameter is computed according to Eq. (1) in the Supplementary Information. The error bars represent 95% confidence intervals for the Warren–Cowley parameter, computed from 40 configurations generated in each training step.

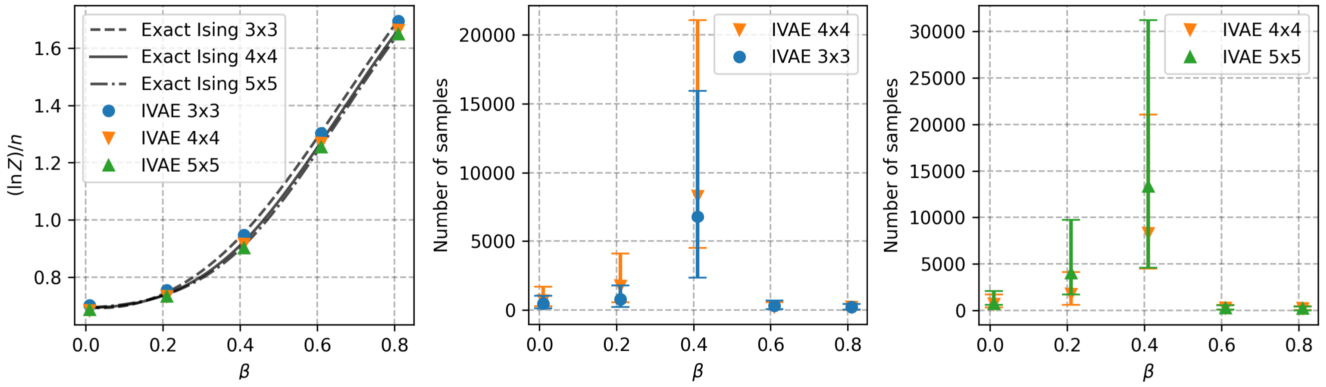

FIG. 5. Experiments using IVAE for the square lattice Ising model (Eq. (2) in the Supplementary Information) on 3×3 (9), 4×4 (16), 5×5 (25) nodes. Left: estimates of $(\ln Z)/n$ (with n the number of nodes) for different values of $\beta = 1/T$. The solid line shows the analytic result. Center: amount of samples required to achieve 99% accuracy by IVAE models for 3×3 and 4×4 Ising systems. Right: amount of samples required to achieve 99% accuracy by IVAE models for 4×4 and 5×5 Ising systems. Error is reported as a 99% confidence interval based on 60 estimations of $\ln Z$.
